# Supplementary material for: HGF-Induced PD-L1 Expression in Head and Neck Cancer: Preclinical and Clinical Findings
Source: Int J Mol Sci. 2020 Nov 20;21(22):8770. doi: 10.3390/ijms21228770 (PMC7699574; doi:10.3390/ijms21228770)
Supplement: Supplementary file 1 [file ijms-21-08770-s001.zip › Supplementary figure/Figure S1 figure caption.pdf]

Figure S1: Increased PD-L1 concentration on cell surface is Met-receptor dependent.

FaDu cells were transfected with two different siRNA constructs specific for the Met receptor (Met siRNA I and II) or a control siRNA. 48 hours after transfection cells were treated with HGF or remained untreated. Cells were subjected to flow cytometry after additional 48 hours using a PE coupled Met specific antibody in (a), (b) and (c), an APC coupled PD-L1 specific antibody in (d), (e) and (f) or the corresponding isotype controls (light colored curves). Panels (a), (b), (d), and (e) show histograms of cells transfected with the indicated siRNAs, (c) and (f) are the median fluorescence of the corresponding histograms shown in (a), (b), (d), and (e) (isotype controls subtracted). One typical result out of six experiments is shown.
