# Supplementary figures and images for: HGF-Induced PD-L1 Expression in Head and Neck Cancer: Preclinical and Clinical Findings
Source: Int J Mol Sci. 2020 Nov 20;21(22):8770. doi: 10.3390/ijms21228770 (PMC7699574; doi:10.3390/ijms21228770)

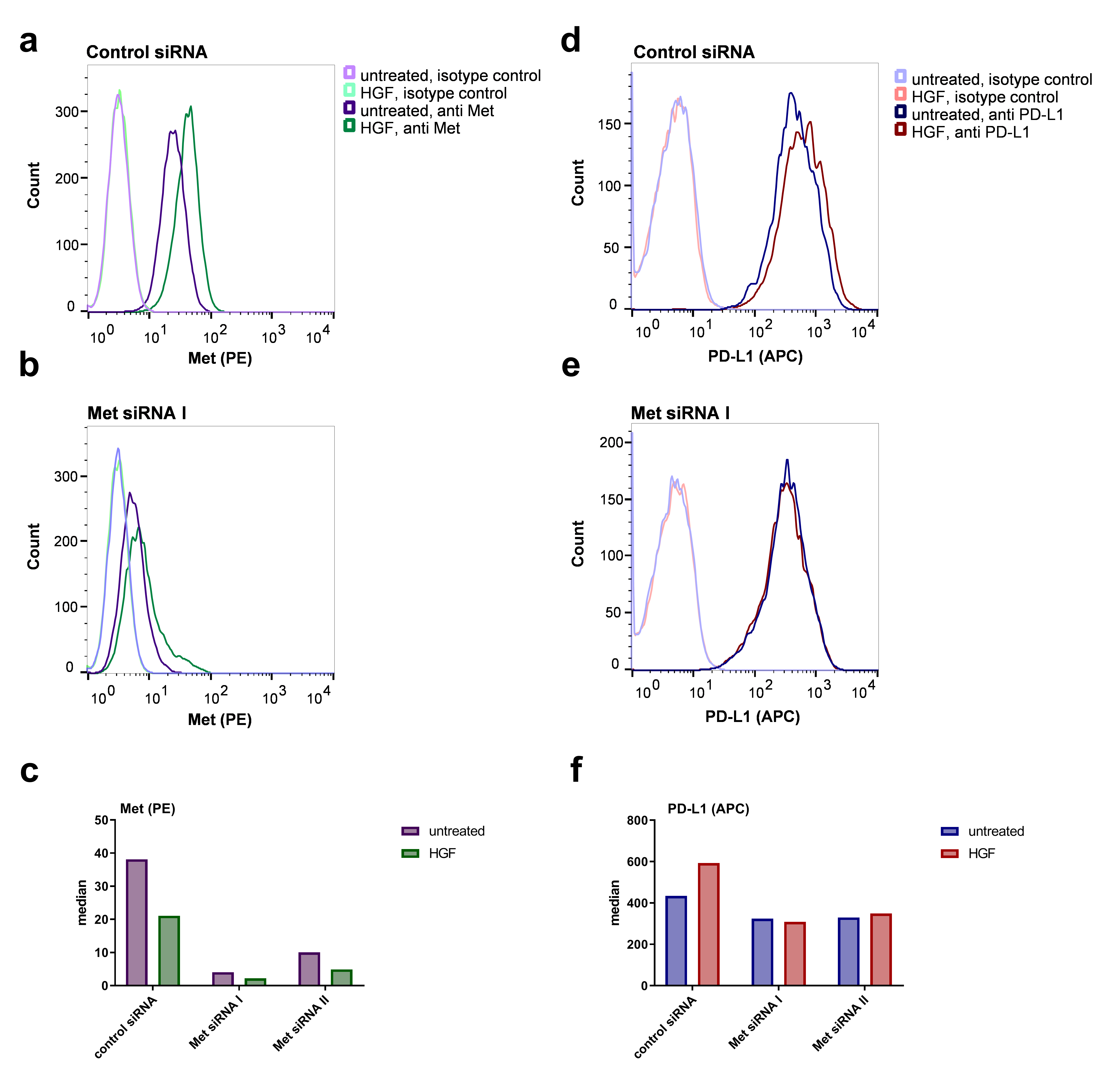

Supplement: Supplementary file 1 [file ijms-21-08770-s001.zip › Supplementary figure/Figure S1.tif]
